# Heat Acclimatization Protects the Left Ventricle from Increased Diastolic Chamber Stiffness Immediately after Coronary Artery Bypass Surgery: A Lesson from 30 Years of Studies on Heat Acclimation Mediated Cross Tolerance

**DOI:** 10.3389/fphys.2017.01022

**Published:** 2017-12-11

**Authors:** Arthur Pollak, Gideon Merin, Michal Horowitz, Mara Shochina, Dan Gilon, Yonathan Hasin

**Affiliations:** ^1^Department of Cardiology, Hadassah Medical Center, Hebrew University of Jerusalem, Jerusalem, Israel; ^2^Department of Cardio-Thoracic Surgery, Hadassah Medical Center, Hebrew University of Jerusalem, Jerusalem, Israel; ^3^Laboratory of Environmental Physiology, Faculty of Dentistry, The Hebrew University of Jerusalem, Jerusalem, Israel; ^4^Department of Rehabilitation, Hadassah Medical Center, Hebrew University of Jerusalem, Jerusalem, Israel

**Keywords:** heat acclimation, heat acclimatization, coronary bypass, diastolic stiffness, cross-tolerance

## Abstract

During the period of 1986–1997 the first 4 publications on the mechanical and metabolic properties of heat acclimated rat's heart were published. The outcome of these studies implied that heat acclimation, sedentary as well as combined with exercise training, confers long lasting protection against ischemic/reperfusion insult. These results promoted a clinical study on patients with coronary artery disease scheduled for elective coronary artery bypass operations aiming to elucidate whether exploitation of environmental stress can be translated into human benefits by improving physiological recovery. During the 1998 study, immediate-post operative chamber stiffness was assessed in patients acclimatized to heat and low intensity training in the desert (spring in the Dead Sea, 17–33°C) vs. patients in colder weather (spring in non-desert areas, 6–19°C) via echocardiogram acquisition simultaneous with left atrial pressure measurement during fast intravascular fluid bolus administration. We showed that patients undergoing “heat acclimatization combined with exercise training” were less susceptible to ischemic injury, therefore expressing less diastolic dysfunction after cardiopulmonary bypass compared to non-acclimatized patients. This was the first clinical translational study on cardiac patients, while exploiting environmental harsh conditions for human benefits. The original experimental data are described and discussed in view of the past as well as the present knowledge of the protective mechanisms induced by Heat Acclimation Mediated Cross-tolerance.

## Introduction

A variety of environmental factors are known to influence cardiovascular morbidity and mortality among which climate and ambient temperature appear to have a considerable effect. Multiple reports from throughout the world suggest a seasonal pattern in the occurrence of acute ischemic syndromes and their complications. Higher environmental temperatures appear to reduce the risk of the occurrence and severity of cardiovascular events (Cech et al., 1977; Marchant et al., 1993; Xu et al., 2013). In contrast, however, in most studies, an increase in the prevalence of acute myocardial infarction and a higher infarction-related mortality rate were observed in winter time (Cech et al., 1977; Ornato et al., 1996; Spencer et al., 1998; Chen et al., 2016). Thus, cold weather, by influencing multiple hemodynamic, rheologic and hematologic variables (e.g., Gordon et al., 1987; Kawahara et al., 1989; Stout and Crawford, 1991; Woodhouse et al., 1993), has been suggested as trigger to acute cardiovascular morbid events.

Animal data have shown that prolonged exposure to moderate ambient heat, heat acclimation (33–34°C, upper limit of the thermoneutral zone-TNZ) induces structural and biochemical changes in the rat heart. These changes are related to the improved systolic and diastolic function and the increased metabolic efficiency observed in heat-acclimated hearts during normoperfusion (Horowitz et al., 1986, 1993; Levi et al., 1993; Levy et al., 1997). Furthermore, heat-acclimated hearts, when compared to non-acclimated hearts, preserve their mechanical and metabolic performance better during hypoperfusion and demonstrate a markedly improved recovery of contractile function and energy stores on reperfusion (Levi et al., 1993; Levy et al., 1997). These beneficial effects of heat acclimation on the ischemic heart are markedly enhanced when rats undergo combined heat acclimation and exercise training (Levy et al., 1997).

The advantages of the trained heat acclimated groups are qualitatively similar to those observed subsequent to sedentary acclimation, but greater in magnitude, on diastolic function in particular (Levy et al., 1997). Thus, in experimental animals, heat acclimation combined with exercise appears to induce efficient protective mechanisms against both ischemic and reperfusion insults. The potential benefits of these protective features, which are currently attributed to “heat acclimated cross-tolerance mechanisms” (Horowitz, 2016) was never evaluated in humans in real clinical settings except for our 1998 pioneering study (Pollak et al., 1998). Here we describe our 1998 collaborative study, attempted to translate the beneficial effects conferred by heat acclimation into a real clinical setting. Notably, the term acclimation describes adaptive changes occurring within an organism in response to experimentally induced changes. When adaptive responses are caused by stressful changes in the natural climate, (e.g., seasonal or geographical) the term acclimatization is used (IUPS Thermal Commission, 2001). The latter terminology was used here for the studied patient.

Cardiopulmonary bypass (CPB) during coronary artery bypass surgery imposes severe myocardial ischemia followed by reperfusion. Both ischemia and reperfusion are known causes of diastolic dysfunction. For example, an intraoperative study using transesophageal echocardiography has shown an increase in diastolic chamber stiffness immediately after coronary artery bypass surgery reflecting worsening diastolic dysfunction (McKenney et al., 1994).

In this investigation we aimed to prospectively study the influence of heat acclimatization with modest exercise training on diastolic dysfunction imposed by CPB in patients with coronary artery disease undergoing coronary artery bypass surgery. We utilized transesophageal echocardiography in conjunction with simultaneous hemodynamic monitoring during fluid challenge to evaluate diastolic function before and immediately after CPB.

## Methods

The study was approved by the Institutional Review Board for human's experimentation of Hadassah University Hospital, Jerusalem, Israel.

Male patients with coronary artery disease scheduled for elective coronary artery bypass operations were enrolled to participate in this study. All patients resided in between zero (sea level) to 700 m elevation. To focus on the effects of CPB and coronary revascularization on diastolic function in a clinically homogenous group we excluded patients for the presence of more than mild (grade 1) mitral or aortic regurgitation, any valvular disease requiring valve replacement, echocardiographic evidence of left-ventricular (LV) hypertrophy, and a significantly reduced LV ejection fraction (less than 45%). Eligible patients were divided into two groups: heat acclimatized (study) group and non-acclimatized (control) group.

### Heat acclimatization combined with exercise training

Eight patients were prospectively and consecutively included in the acclimatized study group as they agreed to leave their home and spend 2 to 3 weeks on the Dead-Sea seashore (lowest point on earth, 410 m below sea level) immediately prior to operation. On location they were accommodated in caravans, devoid of air-conditioning, at an ambient temperature (day-night range) of 17–33°C. These patients exercised twice a day as a group under the supervision of a certified physiotherapist and a physician. An individual training program was developed for each patient. To assess training levels, maximal workload capacity of each patient was determined by a modified “sprint” test (Jennings et al., 1986; Meredith et al., 1990) on a bicycle ergometer, beginning with zero load. The workload was increased every minute by 15 W until further increase was prevented by fatigue. In every case, training exercise load was not increased beyond 65% of maximal heart rate determined by the test. Exercise was terminated earlier if symptoms of chest pain, dyspnea, fatigue and dizziness developed. Each exercise morning session consisted of 5 min. warm-up (stretching exercises of upper and lower extremities), and three stations of aerobic training on a treadmill, stationary ergometer and APT (Active Passive Trainer). The patients exercised between 1 and 5 min at each station. The time for each exercise session was dependent on the time required for each patient to achieve elevated heart rate. After completing each station the patients cooled down until heart rate returned to baseline. Rotation among the 3 aerobic stations was repeated twice. The last 10 min was an extended cool-down period. Afternoon 45 min. session included general upper and lower extremity strengthening exercises, breathing exercise and relaxation. Other than the structured exercise sessions, study patients were free to do as they wished. Patients were operated upon within 3 days after completion of the 14 to 21 day conditioning period.

### Non-acclimatized control group

Eight patients were prospectively matched to those included in the acclimatized group by age, sex, severity of coronary artery disease, diastolic LV dimensions, LV mass and LV systolic function. They were scheduled for elective coronary artery bypass grafting during the same time period as their matched study patients. However, they were not asked to leave their home, thus being exposed to an ambient temperature (day-night range) of 6–19°C. These patients' daily routine was not changed by any means prior to operation.

### Intraoperative protocol

Preparation of patients for open-heart surgery, including endotracheal intubation and general anesthesia was carried out using standard methods. The transesophageal probe was placed after the patient was fully anesthetized. At the time of aortic and venous cannulations, a left atrial line was also placed. While the patient was in steady state, a baseline echocardiogram (M-mode and 2-D, transgastric short-axis view of the left ventricle at mid-ventricular level) was recorded simultaneously with mean left atrial pressure (LAP) measurement. Subsequently, 500 to 1,000 cc of fluid was administered as a fast intravascular bolus over 5 to 10 min; this consisted of crystalloid solution (Standard bypass solution: PlasmaLite® with 2% mannitol). The infusion was continued until the LAP was increased by, at least, 5 mmHg over baseline. Echocardiogram acquisition simultaneous with LAP measurement was continuously recorded until fluid administration ceased. Thereafter, surgical routines were continued uninterrupted throughout the bypass period.

After discontinuation of CPB but before decannulation, while the patient has been fully warmed-up and has re-achieved steady state (i.e., systolic blood pressure of, at least, 100 mmHg and regular sinus or atrially-paced rhythm of less than 100 bpm), a new baseline echocardiogram was recorded simultaneously with mean LAP measurement. Subsequently, 500 to 1,000 cc of fluid was administered as a fast intravascular bolus over 5 to 10 min; this was likely to consist of whole blood and/or plasma but may have been supplemented by crystalloid solution (Standard bypass solution: PlasmaLite® with 2% mannitol and/or Hemacel). This infusion was continued until the LAP was increased by, at least, 5 mmHg over the new baseline. Echocardiogram acquisition simultaneous with LAP measurement was continuously recorded during volume load. Thereafter, surgical routines were continued as usual for decannulation and closure of chest wall. It is important to note that extreme care was taken to assure that the transesophageal echocardiography probe was kept at precisely the same place between the two echograms.

### Data analysis

Off-line echocardiographic analysis was performed with the interpreter being blinded to the group a particular patient belonged to. End-diastolic frames of the LV short axis view were identified. The LV area was traced at end-diastole and related to the corresponding LAP. Traced LV areas of 4 to 7 consecutive cardiac cycles were averaged for each increment of LAP to account for respiratory variations in LV size. LAP measurements were plotted against corresponding mean LV area to generate diastolic pressure-area curves from data obtained before and after CPB, one pair of curves for each patient. To assess changes in LV diastolic properties, LV area was compared at similar left atrial pressures (within 1 mmHg) for the range of LAP changes with volume loading. Thus, for a given LAP, a smaller LV area implies a leftward shift of the LV pressure-volume relationship while a larger LV area suggests a rightward shift of the pressure-volume curve.

### Statistical analysis

In view of the relatively small sample size of both the intervention and control groups of patients, data was expressed as median values and their ranges and non-parametric tests were used for statistical calculations. The Mann-Whitney rank-sum test was used to compare variables between the groups. Proportions were compared by Fisher exact test. The Wilcoxon signed-rank test was utilized in comparing pre- and post-bypass data, with each patient serving as his own control. A *P*-value of less than 0.05 was considered statistically significant.

## Results

Patient characteristics are presented in Table 1. Acclimatized and control groups were well matched for baseline variables (i.e., age, risk factors, LV size and mass) as well as intraoperative parameters (i.e., number of grafts, bypass time, intraoperative body cooling). Acclimatized patients were subject to an average daily temperature of 12°C higher than non-acclimatized patients during the immediate preoperative period (Table 1).

**Table 1:** Baseline and intraoperative variables in acclimatized and non-acclimatized patients.

	**Acclimatized group (*n* = 8)**	**Control group (*n* = 8)**	***P*-value**
Age (years)	65 (44–85)	67 (49–73)	0.88
Hypertension	5/8	4/8	1.00
Diabetes mellitus	4/8	2/8	0.61
LV end-diastolic diameter (mm)	47 (40–63)	48 (42–59)	0.96
LV wall thickness (mm)	10.4 (7.7–11.3)	10.5 (8.4–11.8)	0.87
LV mass (gr.)	199 (136–312)	208 (145–310)	0.87
Number of vessels grafted	3 (2–4)	3 (2–5)	0.88
Cardiopulmonary bypass time (min)	87 (54–121)	96 (57–203)	0.55
Aortic cross-clamp time (min)	57 (31–86)	66 (34–93)	0.23
Systemic cooling temperature (C°)	30 (27–34)	31 (28–34)	0.67
LVEDA before bypass and before loading (cm^2^)	17.02 (10.6–26.1)	19.71 (13.9–28.6)	0.11
LVEDA before bypass and after loading (cm^2^)	20.53 (15.5–29.7)	23.31 (18.5–30.44)	0.23
Ratio of ΔP/ΔA before CPB	1.48	1.49	
Range of day-night temperatures (C°)	17–33	6–19	0.0006

*CPB, cardiopulmonary bypass; LV, left ventricular; LAP, left atrial pressure; LVA, left ventricular end-diastolic area; LVEDA, left ventricular end-diastolic area; ΔP, difference in left-atrial pressure before and after fluid loading; ΔA, difference in left-ventricular area before and after fluid loading Data presented as median (range) or proportion, as needed*.

There was no significant difference in baseline LV end-diastolic area (LVA) between the study and the control groups, both before and after volume loading. The ratio of the difference in LAP to the difference in LVA before and after fluid loading is strongly related to the “slope” of the LV pressure-volume curve. This ratio was virtually identical in both the study and control groups, suggesting no difference in baseline (pre-CPB) diastolic function of acclimatized vs. non-acclimatized patients (Table 1, Figure **2**).

**Figure 1:**
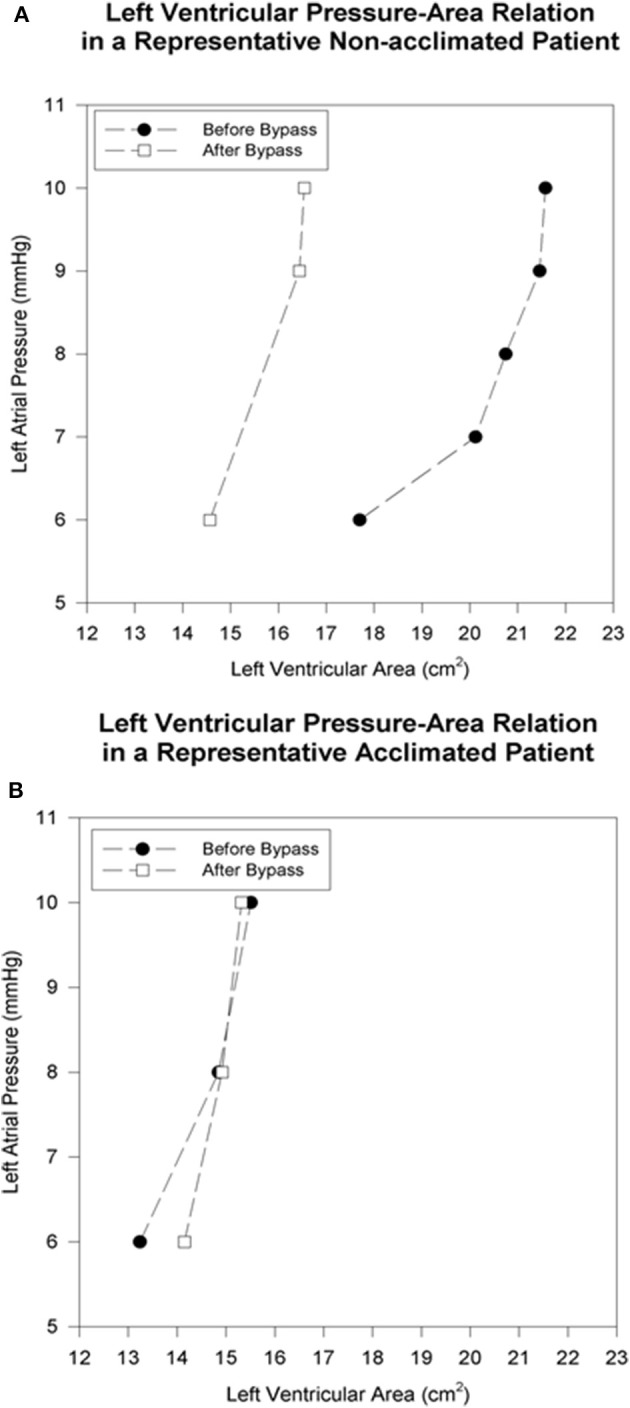
Left ventricular pressure-area relation in representative patients before and immediately after cardiopulmonary bypass. The baseline left atrial pressure of 6 mmHg is progressively increased by volume loading. **(A)** In the non-acclimatized patient, the left-ventricular end-diastolic area is smaller after bypass at each pressure level, as reflected by a leftward shift of the pressure-area relation. **(B)** In the acclimatized patient, the pressure-area relation maintains its position after bypass, without significant change in left-ventricular end-diastolic area at comparable pre- and post-bypass pressures.

**Figure A1:**
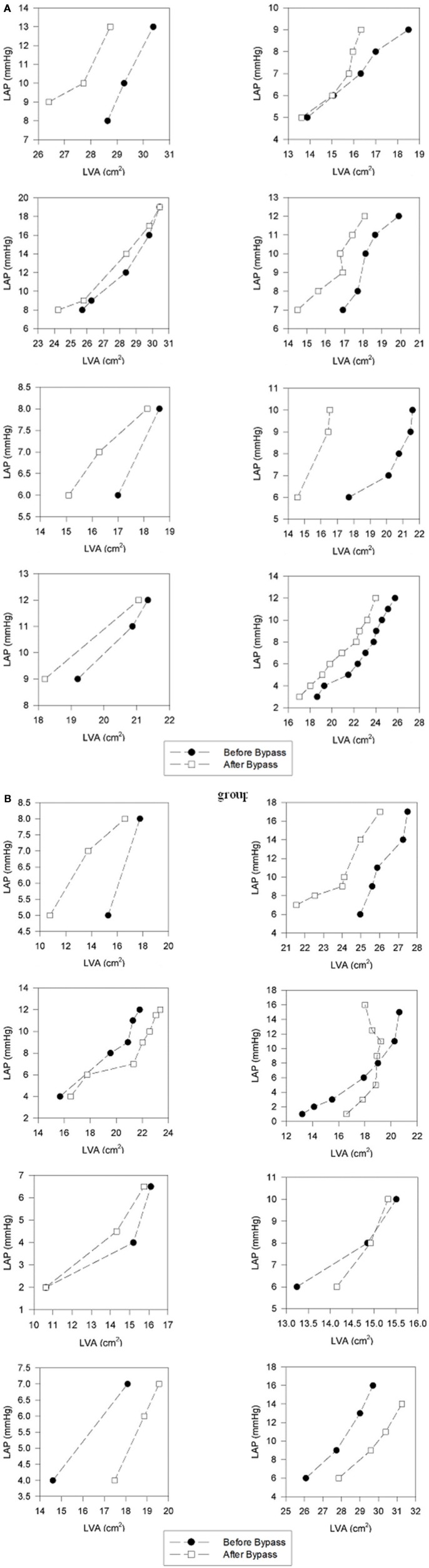
Individual LV Left ventricular pressure-area relations pre- and post-bypass surgery. **(A)** Individual LV pressure-area relation pre- and post-bypass in the control patient; **(B)** Individual LV pressure-area relation pre- and post-bypass in acclimatized patients.

Diastolic pressure-area curves before and after CBP in a representative patient from each group is depicted in Figure 1 (individual diastolic pressure-area curves of all patient appear as in the Appendix, Figure A1).

**Figure 2:**
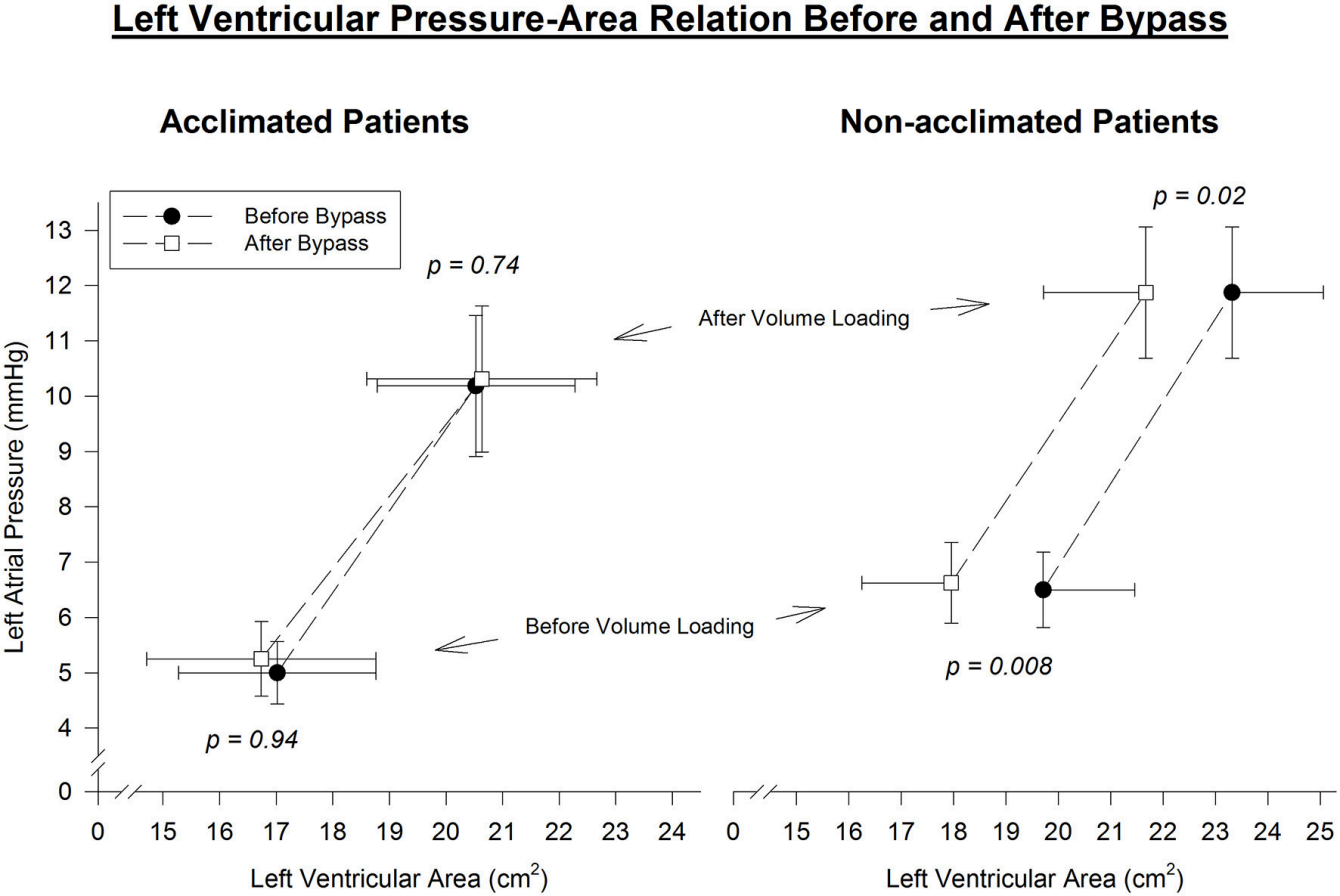
Left ventricular end-diastolic pressure-area relation before and immediately after cardiopulmonary bypass. Pre- and post-bypass left-ventricular area is compared at similar left-atrial pressure throughout the range of volume manipulation. A parallel leftward shift of the pressure-area curve is evident in non-acclimatized patients, consistent with reduced left-ventricular diastolic distensibility after bypass. In contrast, the slope and position of the pressure-area curve is maintained after bypass in acclimatized patients, indicating no significant change in left-ventricular diastolic properties.

Left ventricular area decreased after CPB in all control patients at comparable preloads. Before fluid loading, at mean left atrial pressure 6.5 mmHg, there was a 1.8 ± 0.3 cm^2^ decrease in LVA (*p* = 0.008). After fluid loading, at mean left atrial pressure 11.9 mmHg, there was a 1.7 ± 0.6 cm^2^ decrease in LVA (*p* = 0.02). Thus, a distinct leftward shift of the diastolic pressure-area curve was noted in non-acclimatized patients (Figure 2).

However, in patients who underwent heat acclimatization, no change in left ventricular area measured at comparable preloads before and after CPB was noted. Both before and after fluid loading, at mean left atrial pressures of 5 and 10.3 mmHg, respectively, mean left ventricular area pre-bypass was similar to left ventricular area post-bypass. Thus, a virtual overlap of the pre- and post-CPB diastolic pressure-area curves was noted (Figure 2).

## Discussion

This study was the first clinical research, which exploited environmental stressor to improve human function/health by establishing controlled clinical setting, using a model of ischemia and reperfusion during coronary artery bypass surgery. We showed that patients undergoing “heat acclimatization combined with exercise training” were less susceptible to ischemic injury, therefore expressing less diastolic dysfunction after CPB compared to non-acclimatized patients. The relative contributions of heat vs. exercise were not studied. However, based on contemporary knowledge, this issue will be discussed. CPB imposes a multi-factorial burden on the myocardium resulting, among other things, in diastolic dysfunction. McKenney et al. (1994) have shown that a leftward shift in the LV diastolic pressure-area curve occurs immediately after CPB in patients undergoing coronary artery bypass surgery. This increase in LV diastolic chamber stiffness may be related to several mechanisms including ischemia, cooling and interstitial edema. Subsequent work done by the same group suggests that the deterioration in diastolic properties persists into the post-operative period for at least 3 h (Ekery et al., 2003) and may indeed have clinical relevance in the post-operative hemodynamic management of these patients.

Without exception, there was a decrease in LV distensibility in each of our control patients immediately after CPB. This is in complete accordance with the previous work cited (McKenney et al., 1994; Ekery et al., 2003). It supports the universal applicability of our group of patients (i.e., this is not an unusual or improperly selected patient population). In contrast, pre-bypass diastolic LV distensibility did not changed in the acclimatized patient group. Both end-diastolic area and the slope of the pressure-area curve were practically identical before and after bypass, suggesting preservation of the pre-bypass diastolic properties throughout all loading conditions. Thus, our results indicate that 2 to 3 weeks of exposure to heat in combination with modest exercise training abolished the worsening in diastolic chamber distensibility, which is commonly related to bypass operative techniques.

Environmental factors can influence the structure and physiological characteristics of the myocardium as well as its ability to withstand ischemia and reperfusion. In animal experiments, heat acclimation with/without exercise proved to have a significant cardioprotection and to enhance recovery from ischemia/reperfusion insults. The beneficial effects were manifested by an improved chamber compliance (Horowitz et al., 1986) under normoxic conditions as well as greater systolic pressure generation with lower O_2_ consumption (Horowitz et al., 1993; Levi et al., 1993; Levy et al., 1997; Eynan et al., 2002; Horowitz, 2003). Upon global ischemia and reperfusion insults, there was a delayed appearance of ischemic contracture, and better recovery of diastolic and systolic functions (Levi et al., 1993; Levy et al., 1997). This was concomitant with ATP preservation during ischemia in the heat acclimated hearts with and without exercise. This preservation of ATP, in both sedentary and trained hearts, implied the predominant contribution of the prolonged heat exposure *per se* on the recovery of diastolic function (Levi et al., 1993). To-date, our knowledge of the mechanism of heat acclimation mediated cross tolerance and cardioprotection has been largely advanced. There is heat acclimation induced decrease in Ca^2+^ sensitivity, thus the heart is less susceptible to Ca^2+^ overload (Kodesh et al., 2011). Concomitantly, gene chip analysis demonstrated profound upregulation of Nebulin, a giant, actin-binding protein that stabilizes the thin filaments for length maintenance and is also associated with Ca^2+^ sensitivity, increasing contractile strength and efficiency (Tetievsky et al., 2014). Collectively, these changes most likely underlie the qualitative alterations in the electrical component of the excitation-contraction coupling (Kodesh et al., 2011) and the structural modifications that enhance cardiac compliance and improve the ability to cope with the peripheral volume overload seen after heat acclimation or heat stress (Horowitz, 2003). Both HSP72 and the hypoxia transcriptional factor HIF-1α reserves are constitutively elevated and play an important role in cytoprotection under ischemic conditions (Maloyan et al., 1999, 2005; Horowitz, 2016).

Recently, heat acclimation induced cross tolerance was “navigated” to thermotherapy of vascular diseases. Brunt et al. (2016a,b) showed that, relative to a sham group which participated in thermoneutral water immersion, passive heat therapy for 8 weeks increased flow-mediated dilatation, reduced arterial stiffness, reduced mean arterial and diastolic blood pressure and that the vasodilation was NO dependent. Although these data are linked to major blood vessels rather than to the heart, the outcome fits with our postulate, based on animal studies, that heat *per se* enhances cardiovascular compliance, leading to improved exercise capacity and diastolic function in the ischemic heart.

Our recent studies shows that heat acclimation involves epigenetic control of gene expression (Tetievsky and Horowitz, 2010) and includes metabolic switch leading to mitochondrial upregulation of PDK1 and a shift to enhanced glycolytic ATP utilization (Alexander-Shani et al., 2017).

The cross-tolerance between heat acclimation and ischemic injury appears to be a long-lasting effect, for at least 2 to 3 weeks (Cohen O, PhD Thesis, The HU; Eynan M, PhD Thesis, The HU; Horowitz M, personal communication and Arieli et al., 2003).

Increasing number of studies emphasize the role of exercise training in cardioprotection. However, the knowledge of the impact of exercise training on ventricular diastolic function/dysfunction is rather limited and controversial. Bhella et al. (2014) demonstrated that lifelong committed exercise (5–6 sessions/week) prevented decreased compliance in aged healthy subject while Fujimoto et al demonstrated no training effects on compliance unless Alagebrium, which reverses stiffness of blood vessels wall was taken as well. Ades et al. (2013) in their meta-analysis on exercise and cardiac rehabilitation report inconsistencies and reciprocal effects regarding exercise impact on chamber and aortic compliance. Collectively, however, exercise intensity above 65% for at least 2 mo, was needed to impact diastolic function and aortic remodeling. Diastolic function in elite endurance athletes, however, correlated enhanced distensibility with exercise (Levine et al., 1991). In cardiac rehabilitation, exercise intensity above 65% of maximal heart rate determined by sprint test was needed to impact diastolic function or aortic remodeling.

We thus postulated that in our patients undergoing “heat acclimatization combined with exercise training” the heat exposure *per se* mitigates diastolic dysfunction following ischemia-reperfusion. This group would be less susceptible to ischemic injury not only because of changes in elasticity, which likely impact on diastolic dysfunction after CPB compared to non-acclimatized patients, but also because of better energy state and large reserves of cytoprotective proteins.

### Study limitations

A major limitation of this study is that the control group was assigned to “non-committed" to exercise group, which does not allow unequivocal conclusion regarding the relative contributions of heat and endurance exercise to protect post operation diastolic function. However, the low level of exercise in the acclimatized group, together with recent findings on the impact of sedentary thermotherapy support our conclusion that heat exposure *per se* is a major contributor to the results obtained.

A point of note is the question of whether the slight hyperbaric pressure in the Dead Sea has an impact on cardiac stiffness. Abinader et al. (1999), in a pilot study, measuring wall motion in patients with coronary artery disease prior to and 5 days after descending to the dead sea. These subjects demonstrated ~8% decrease WMSI (wall motion score index) vs. their WMSI under normobaric pressure. Matched healthy controls did not show changes in WMSI under similar conditions. The Dead Sea area is known for its high ambient temperatures, suggesting perhaps that high ambient temperature was one beneficial parameters in the improvement observed in these patients. No conclusion statements by the authors was provided.

This work is too small to assess the importance of heat acclimatization on morbidity/mortality after coronary artery bypass surgery. The extension of the deleterious effect of bypass into the post-operative period supports the assumption that enhancement of diastolic function immediately after bypass may eventually prove to be beneficial to improve operative results.

## Conclusion

This investigation is the first clinical study supporting the experimental evidence for a protective effect of heat acclimatization combined with modest exercise training against diastolic dysfunction imposed by ischemic/reperfusion insults. Although this study could not distinguish the relative contribution of each factor on the gained benefit, we believe that this effect is real and chronic heat exposure plays a major role in it. Further clinical studies are justified to expand on this preliminary data and evaluate the longevity of this effect as well as its relevance to the clinical outcome of patients undergoing coronary artery bypass surgery.

## Ethics statement

This study was carried out in accordance with the recommendations of Hadassah committee for Human's experimentation with written informed consent from all subjects. All subjects gave written informed consent in accordance with the Declaration of Helsinki. The study was approved by the Institutional Review Board for human's experimentation of Hadassah University Hospital, Jerusalem, Israel.

## Author contributions

AP: Ecocardiology, interpretation of the results, drafter the paper and the Figures; GM: coronary by-pass surgery; MH: conception of the study, Interpretation of the results in light of the current knowledge of the topic, drafted the paper; MS: Organization of exercise and physiotherapy; DG: Ecocardiology; YH: Conception of the study, Interpretation of the results.

### Conflict of interest statement

The authors declare that the research was conducted in the absence of any commercial or financial relationships that could be construed as a potential conflict of interest.

## Abbreviations

CPB: cardiopulmonary bypass
LV: left ventricular
LAP: left atrial pressure
LVA: left ventricular end-diastolic area.
